# Clinicopathological characteristics, diagnosis, treatment, and outcomes of primary gastric adenosquamous carcinoma

**DOI:** 10.1186/s12957-015-0554-1

**Published:** 2015-04-02

**Authors:** Haining Chen, Chaoyong Shen, Rui Yin, Yuan Yin, Jiaju Chen, Luyin Han, Bo Zhang, Zhixin Chen, Jiaping Chen

**Affiliations:** Department of Gastrointestinal Surgery, West China Hospital, Sichuan University, 37 Guoxue Alley, Chengdu, 610041 Sichuan China; Department of Pathology, West China Hospital, Sichuan University, 37 Guoxue Alley, Chengdu, 610041 Sichuan China; Intensive Care Unit, West China Hospital, Sichuan University, 37 Guoxue Alley, Chengdu, 610041 China

**Keywords:** Adenosquamous carcinoma, Clinicopathologic, Diagnosis, Prognosis, Stomach neoplasms

## Abstract

**Background:**

Primary gastric adenosquamous carcinoma (ASC) is a rare subset of ASC. This study aims to investigate the clinicopathological features, diagnosis, treatment, and outcomes of primary gastric ASC.

**Methods:**

The medical records of 13 consecutive patients with primary gastric ASC between January 2010 and July 2014 from a single institutional database were reviewed.

**Results:**

Male predominance was observed (M/F = 10/3) among the patients, and their median age was 62 years (range: 43 to 79 years). The primary lesions were most often found in the upper third of the stomach, with a median tumor size of 5 cm (range: 2.25 cm to 10.5 cm). Ten patients underwent radical resections (R0 resection, 76.9%), while three patients had palliative resections (R1/R2 resection, 23.1%). Twelve patients had lymph node metastasis at the time of surgery. Adenocarcinoma and squamous cell carcinoma components in lymph node were found in eight and two cases, respectively, while two patients had both squamous cell carcinoma and adenocarcinoma components. In terms of the TNM staging system, stages IIB, IIIA, IIIB, IIIC, and IV were detected in 2 (15.4%), 2 (15.4%), 1 (7.7%), 5 (38.5%), and 3 (23.1%) patients, respectively. The median follow-up period was 22 months (range: 5 to 52 months); during which, four patients were still alive and eight patients died because of tumor progression. The 1-, 2-, and 3-year survival rates were 76.9%, 46.2%, and 15.4%, respectively.

**Conclusions:**

Primary gastric ASC has a very poor prognosis, and both squamous cell carcinoma and adenocarcinoma components have distant metastasis potential.

## Background

Adenosquamous carcinoma (ASC) is a rare entity that occurs throughout the entire alimentary tract [1-3]. The incidence of primary gastric ASC is uncommon, accounting for less than 1% of the total gastric carcinomas worldwide [4,5] and mostly affecting Asians. Primary gastric ASC exhibits early tumor progression and poorer prognosis than the typical gastric adenocarcinoma [4]. The biological behavior of ASC is usually determined by the adenocarcinoma component [6,7]. Patients with gastric ASC have variable clinical symptoms, some of which are identical to those of other types of gastric tumors.

Given the rarity of these carcinomas, most literatures are based on case reports [2,4,5,7-11]. The histogenesis of these tumors has been debated considerably, and clinical therapies and prognosis of ASC have not been well established to date [12]. Therefore, in the current study, we incorporated the data of 13 consecutive patients with primary gastric ASC who underwent surgery at a single medical institution. We investigated the clinicopathological features, diagnosis, treatment, and survival outcomes of these patients to contribute to deeper knowledge on this tumor and provide additional assistance for ASC management.

## Methods

### Patient selection

Thirteen patients who were surgically treated and pathologically diagnosed with primary gastric ASC at the Department of Gastrointestinal Surgery, West China Hospital, Sichuan University from January 2010 to July 2014 were retrospectively identified. Contrast-enhanced computed tomography scan of the chest and abdomen, electronic gastroscopy, lung function, renal and liver function, and others were routinely performed preoperatively. All surgical samples were reviewed by a senior pathologist from our institution. ASC diagnosis was confirmed when the characteristics of coexistence of squamous cell carcinoma (SC) and adenocarcinoma components (AC) were identified, with SC accounting for at least 20% to 25% [13,14]. Patients diagnosed with non-gastric ASC having incomplete medical records and did not attend the follow-up examination were excluded from this study. The Institutional Review Board and Ethics Committee of the West China Hospital of Sichuan University informed that an ethical review was not needed for this retrospective study.

### Surgery and immunohistochemical examination

All patients underwent laparotomy with general anesthesia; surgical procedures were performed according to the criteria of the Japanese Gastric Cancer Association [13], including subtotal gastrectomy or total gastrectomy and D2 lymphadenectomy or greater D2 lymphadenectomy. Operation consent was obtained from each patient who underwent surgical resection. Two patients underwent proximal gastrectomy with gastric tube reconstruction. The specimens for histological examination were fixed in 10% buffered formalin and stained immunohistochemically for carcinoembryonic antigen (CEA), cytokeratin (CK) 5/6/7, p63, chromogranin A (CgA), and synaptophysin (Syn).

### Data collection and follow-up

The parameters collected from the medical records and pathological reports included demographic data, clinical manifestation at the time of diagnosis, hospital stay, surgical data, tumor size and location, pathological data (Borrmann types, depth of invasion, lymphatic metastasis, TNM stages, and so on.), immunohistochemical staining, postoperative tumor recurrence, and so on. Tumor TNM stages were graded according to the criteria of the Seventh Edition of the American Joint Committee on Cancer Staging Manual [15]. Overall survival time was calculated from the time of surgery to death or until the last follow-up. Follow-up was conducted through office visit, telephone, or outpatient clinic visit from September 2014 to October 2014.

## Results

### Patient characteristics

The clinicopathological data of the ASC patients are summarized in Tables 1 and 2. This entire cohort is comprised of 10 males and 3 females, with a male-female ratio of 3.3:1. The ages of the patients ranged from 43 to 79 years (median: 62 years). A preoperative biopsy showed ASC in only two patients. The primary lesions were mostly found in the upper third of stomach, with a median tumor size of 5 cm (range: 2.25 to 10.5 cm). Borrmann types I, II, and III carcinoma were observed in 1 (7.7%), 6 (46.2%), and 6 (46.2%) cases, respectively. The clinical symptoms were identical to those of other types of gastric tumors, with epigastric pain, dysphagia, and acid regurgitation as the main clinical manifestations. The mean hospital stay length was 19.3 ± 6.1 days. Two patients (numbers 2 and 5) had type 2 diabetes mellitus, while one patient (number 5) was confirmed with liver cirrhosis during the operation.

**Table 1:** **Clinicopathological characteristics and demographical data of 13 patients with primary gastric ASC**

**Case number**	**Gender**	**Age**	**Site**	**Size (cm)**	**Borrmann type**	**Hospital stay (days)**	**Operation**	**Postoperative complication**	**Adjuvant therapy**
1	M	51	U	2.5*2	II	29	Radical	Abdominal infection	Yes
2	F	66	M	11*10	III	28	Palliative	-	No
3	M	63	U	7*6	III	21	Radical	-	No
4	M	43	L	6*5	II	11	Radical	Pulmonary infection	Yes
5	M	79	U	7*5	III	27	Palliative	Ileus	Yes
6	M	62	U	8*4	II	21	Radical	-	No
7	M	47	L	5*5	II	11	Radical	-	No
8	F	70	U	4*4	I	18	Radical	-	No
9	M	63	L	5*5	III	17	Radical	-	Yes
10	M	73	L	4*3	III	13	Radical	Digestive bleeding	No
11	M	56	U	6*3	II	16	Palliative	-	Yes
12	F	50	M	3*3	II	22	Radical	-	Yes
13	M	61	U	5.5*4	III	17	Radical	-	No

*Abbreviations*: *ASC* adenosquamous carcinoma, *M* male, *F* female, *U* upper third of stomach, *M* middle third of stomach, *L* lower third of stomach.

**Table 2:** **Pathological characteristics and survival outcomes of 13 patients with primary gastric ASC**

**Case number**	**TNM stage**	**Depth of invasion**	**Metastasis rate of LN**	**Distant metastasis**	**Proportion of SCC**	**Metastatic components in LN**	**Recurrence/metastasis (months)**	**Outcomes/period (months)**
1	IIIA	T4a	2/24	M0	40%	AC	Liver (7)	Death (26)
2	IV	T4b	4/29	Spleen/pancreas	25%	AC	Peritoneal cavity (0)	Death (5)
3	IIIC	T4a	16/63	M0	65%	AC and SC	Anastomosis (2)	Death (7)
4	IIIB	T3	8/32	M0	25%	AC	Peritoneum (17)	Death (31)
5	IV	T4b	4/27	Liver	35%	AC	Liver (0)	Alive (15)
6	IIB	T3	0/12	M0	40%	Absent	Peritoneum (21)	Death (34)
7	IIIC	T4a	27/32	M0	30%	AC and SC	Lung (13)	Death (18)
8	IIIA	T4a	2/19	M0	75%	AC	Absent (-)	Alive (32)
9	IIIC	T4a	11/19	M0	60%	SC	Liver (10)	Alive (41)
10	IIB	T2	4/21	M0	20%	AC	Absent (-)	Alive (52)
11	IV	T3	9/29	Liver	35%	AC	Liver (0)	Death (13)
12	IIIC	T4b	8/24	M0	25%	SC	Liver (9)	Death (22)
13	IIIC	T4a	11/28	M0	45%	AC	Peritoneum (5)	Death (11)

*Abbreviations*: *ASC* adenosquamous carcinoma, *LN* lymph nodes, *AC* adenocarcinoma components, *SC* squamous cell carcinoma components.

### Surgical and adjuvant treatment

All 13 ASC patients were surgically treated through abdominal approach, including D2 lymphadenectomy or greater D2 lymphadenectomy. Ten patients underwent radical resections (R0 resection, 76.9%), while the other three patients had palliative resections (23.1%), including multiple liver metastasis (number 11), liver metastasis with cirrhosis (number 5), and involvement of pancreas/spleen (number 2). Total, proximal, and distal gastrectomy was conducted in 3, 6, and 4 cases, respectively. Two patients underwent gastric tube reconstruction after proximal gastrectomy. In terms of the TNM staging system, stages IIB, IIIA, IIIB, IIIC, and IV were detected in 2 (15.4%), 2 (15.4%), 1 (7.7%), 5 (38.5%), and 3 (23.1%) patients, respectively. Adjuvant therapies were routinely recommended for patients with palliative resection, and stage II, III, or IV. Chemotherapy regimens for squamous carcinoma should be conducted when SC component becomes a dominant in tumor tissue. A total of six patients received adjuvant therapy. Three patients were treated with chemotherapy of SOX regimen for five cycles (numbers 1, 9, and 11), while the other three patients received FOLFIRI for three cycles (number 5), FOLFOX for seven cycles (number 4), and Gimeracil and Oteracil Porassium capsules for four cycles (number 12). Two (numbers 1 and 9) of the six patients also received abdominal radiotherapy (GTV 60.2GY/28f, CTV 50.4GY/28f).

### Pathological and immunohistochemical examination

**Figure 1:**
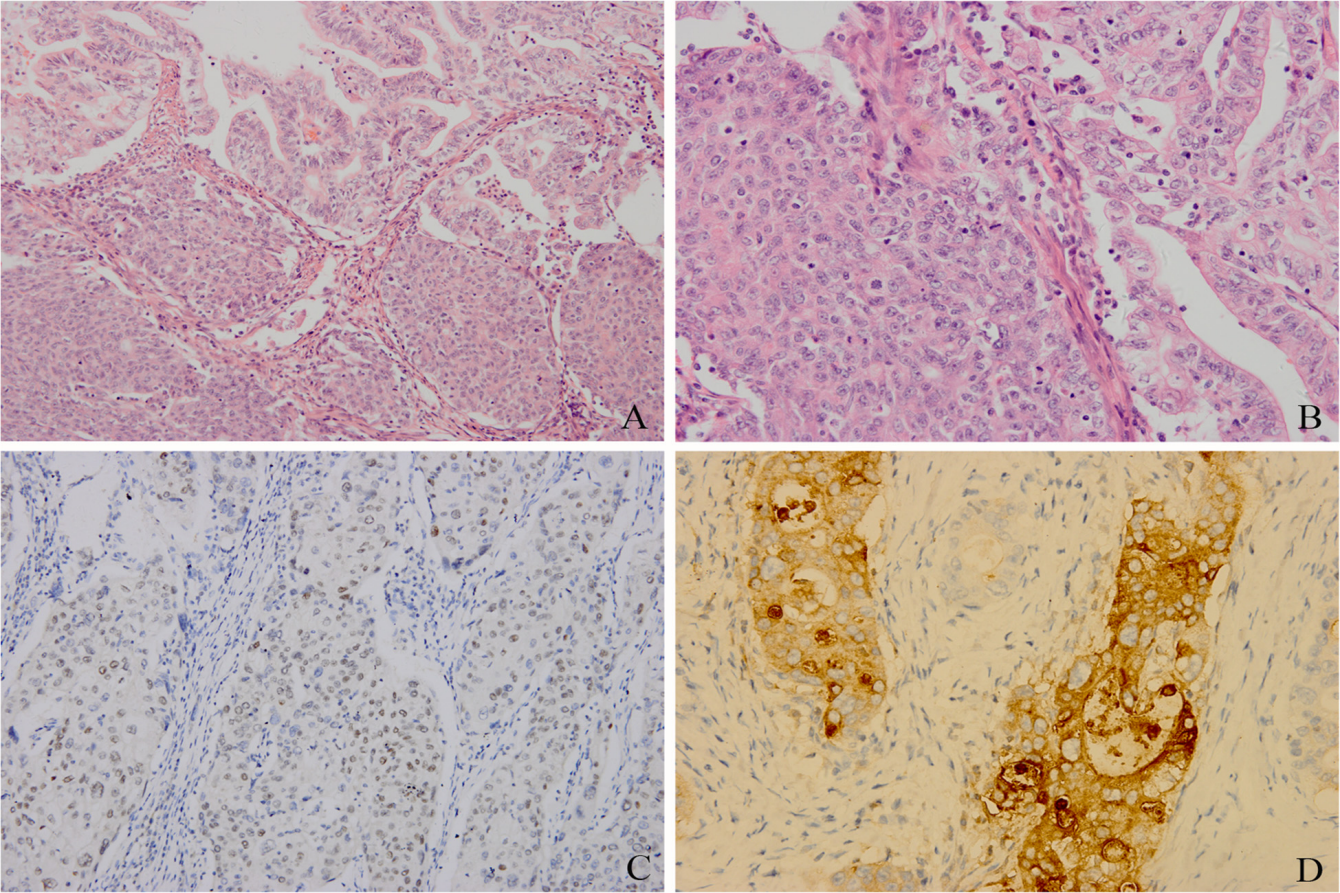
**ASC characterized as a mixture of SC and AC.** Both squamous cell carcinoma and adenocarcinoma components were found (**A**: hematoxylin-eosin staining, ×200; **B**: hematoxylin-eosin staining, ×400); Immunohistochemistry of p63 protein that was positive in the squamous cell carcinoma area (**C**: ×400), and positive staining for adenocarcinoma cell with CEA (**D**: ×400).

**Figure 2:**
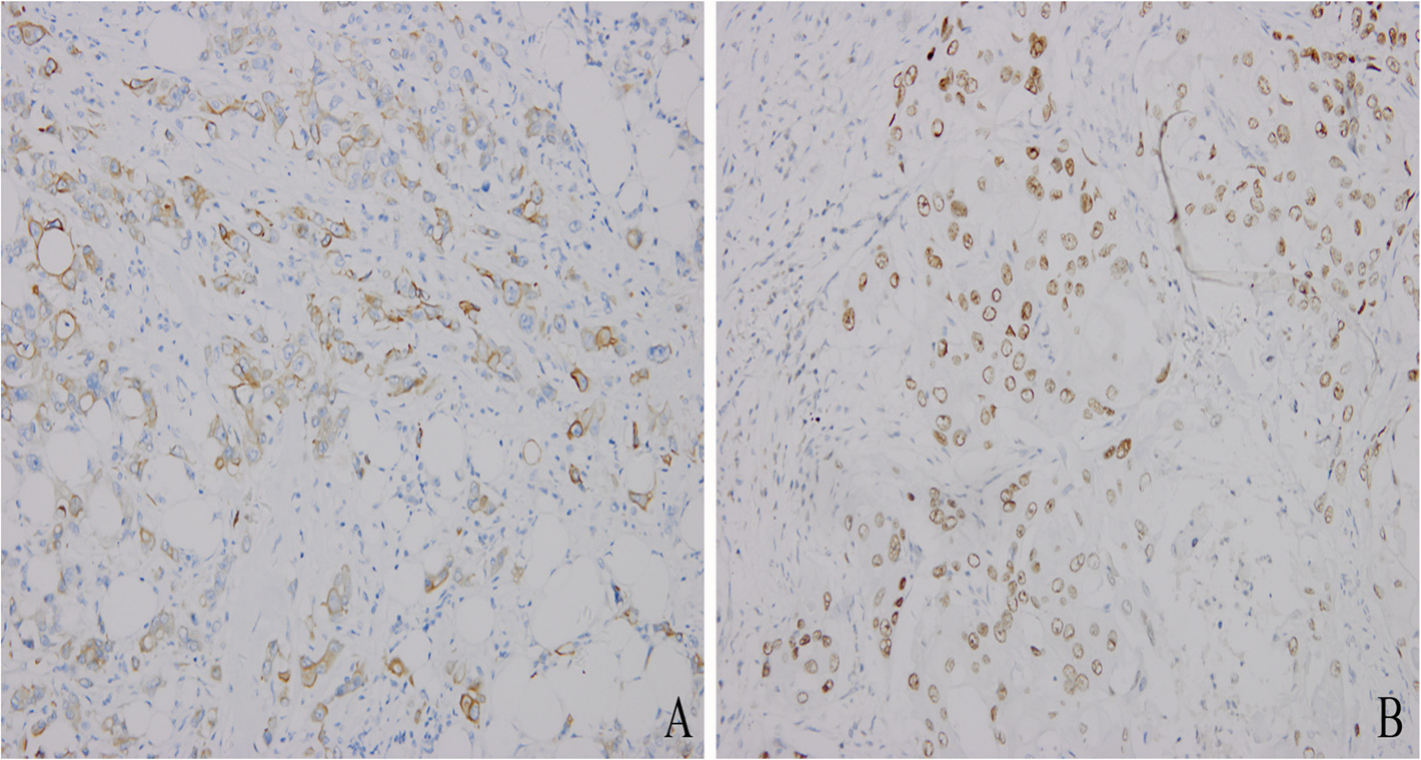
**Squamous cell carcinoma and adenocarcinoma components were found in the lymph node metastasis in the same patient.** Adenocarcinoma component, positive for CK7 (**A**: ×400); and squamous component, positive for p63 (**B**: ×400).

Table 2 shows the pathological characteristics of the 13 primary gastric ASC patients. ASC was characterized as a mixture of SC and AC (Figure 1). Most squamous cell carcinoma components had the characteristics of individual cell keratinization, keratin pearl, or intercellular bridge, among others. Seven patients underwent immunohistochemical examination. Both CEA and CK7 were positive in all AC of tumor or AC of metastatic lymph node; however, SC with these two antibodies were weakly positive in one and three cases, respectively. P63 expression was positive in the squamous cell carcinoma component in six patients but negative in adenocarcinoma components. No patient had positive expression of CgA and Syn. The tumor invaded the visceral peritoneum (T4a) and adjacent tissues (T4b) in six and three cases, respectively. A total of 359 lymph nodes were moved; among which, 106 had metastases. Adenocarcinoma and squamous cell carcinoma components in lymph node were found in eight and two cases, respectively, while two patients had both squamous cell carcinoma and adenocarcinoma components (Figure 2).

### Survival outcomes

Follow-up was carried out in all 13 patients, with a median of 22 months (range: 5 to 52 months). Four patients were still alive, whereas eight cases died because of tumor progression. Moreover, one patient (number 13) died because of chronic respiratory failure. All but two patients with TNM stages IIIA and IIB experienced tumor recurrence and/or metastasis, with a median time of progression of 7 months. The most common metastases were detected in the liver of five patients, followed by peritoneum in three cases. Meanwhile, one patient (number 9) with liver metastases underwent radiofrequency ablation. The median overall survival time and time to progression were 22 and 9 months, respectively. A trend for better survival was observed in patients treated with adjuvant therapy (median survival time: 24 months *vs.* 18 months). The 1-, 2-, and 3-year survival rates were 76.9%, 46.2%, and 15.4%, respectively. Given the limited sample size, analysis of the relevant prognosis factors of ASC was not attempted.

## Discussion

Adenocarcinoma is the most common malignant tumor of the stomach; primary gastric ASC is a rare clinical entity. ASC of the stomach has gained a great deal of interests because of its unsolved histogenesis; its clinicopathological and immunohistochemical characteristics are poorly understood [7,13]. Rolleston and Trevor first reported on gastric ASC in 1905. Notably, the majority of patients with ASC were diagnosed at an advanced stage, with high-grade malignancy and poor prognosis [2,4]. ASC occurrence is more frequent in the proximal stomach among middle-aged and aged populations, as well as among Asian men [1,5,16]. In the current study, the male to female ratio was 3.3:1. Occurrence is mainly observed in the upper third of the stomach among patients with median age of 62 years, which is in agreement with previous reports. The clinical presentations of gastric ASC can be variable but are basically identical to those of other types of gastric cancer, and presented mainly as epigastric pain [4,7].

According to the World Health Organization classification, ASC consists of a mixture of adenocarcinoma and squamous cell carcinoma components. However, the histogenesis of SC remains controversial. The following hypotheses have been proposed concerning its most likely origin [1,4,8,17-19]: 1. Foci of heterotopic squamous epithelia in the gastric mucosa; 2. Stem cell of the gastric mucosa that differentiated toward both cellular lines; 3. Metaplastic transformation of an adenocarcinoma or benign squamous gastric epithelia; 4. Endothelial cells differentiated toward squamous elements; and 5. Collision of an adenocarcinoma and a squamous cell carcinoma. Currently, the hypothesis that SCs are derived from adenocarcinoma is favored by many scholars. ASC has one obvious transition area between the two components and adjoined adenocarcinoma components. The positive expression of CK7 and CEA in SC from the available data supports the hypothesis that SC originated from adenocarcinoma [1,19]. Moreover, SC in ASC may differentiate into those that resemble pure squamous cell carcinoma or those with totally different biological behaviors [17]. In this study, weak positive expression of CEA and CK7 in SC were observed with one and three cases, respectively. Hence, more studies are required to confirm the histogenesis of gastric ASC.

Macroscopically, the majority of ASCs observed are Borrmann types II and III, whereas types I and IV are rare [17,20]. Diagnosed ASC is usually accompanied by distant metastasis, such as peritoneal dissemination, lymph node metastasis, and liver metastasis [4-6]. As shown in the present series, the most common site of metastasis is the liver, followed by peritoneal dissemination. We also found that both SC and AC have the potential for distant metastasis, similar to those reported in other publications [1,17]. ASC is usually found deep into the muscular layer, with more aggressive clinicopathological characteristics. The findings in this study show that the tumor invaded the visceral peritoneum (T4a) or adjacent tissues (T4b) in six and three cases, respectively. Most patients were diagnosed at more advanced stages. A noteworthy standpoint was raised by Bansal *et al*. [7], who considered that the biological behaviors may be determined by the AC in ASC of the stomach. Meanwhile, several studies have shown that the biological behaviors of ASC are usually similar to those of an aggressive adenocarcinoma [6,18,21]. However, a convincing conclusion cannot be reached based on a case report or observation with a small number of samples.

No standard treatment for primary gastric ASC has been established because of its rarity. At present, complete surgical resection to achieve R0, including resetting the proximity of organs or directly surrounding normal tissues as necessary, remains the main curative method for all malignant tumors. Regrettably, the therapeutic outcomes of ASC are unsatisfactory, and most patients exhibit a very rapid deteriorating course within a few months postoperatively [4,7]. Patients who received adjuvant chemotherapy had better outcomes. S-1 monotherapy, cisplatin, fluoropyrimidine, docetaxel, and irinotecan reportedly improve survival times, but still with poor prognosis [1,5,10,22]. Squamous carcinoma is sensitive to radiotherapy; hence, postoperative radiotherapy can also be used as one of the comprehensive treatments for ASC. As described previously, patients with cervical ASC derive relatively better survival benefits from adjuvant radiotherapy [23,24]. In our study, a trend for better survival in patients treated with adjuvant therapy was observed (median survival time: 24 months *vs*. 18 months). Nevertheless, in an effort to gain confirmation of this finding and establish the standard therapeutic approaches, more data concerning gastric ASC should be collected.

The prognosis of ASC has been discussed in previous reports. ASC is more aggressive than pure adenocarcinoma. The median survival time of gastric ASC ranges from 12 to 22 months, and the 5-year survival rate is 10% [1,5]. A similar survival result of esophageal ASC was reported by Chen *et al* [14]. Their study showed that the median survival time was 21 months, and the 1-, 3-, and 5-year overall survival rates were 67.5%, 29.4%, and 22.9%, respectively. By contrast, cervical ASC patients seem to have a relatively better outcome [23]. In the current study, the median overall survival time and time to progression were 22 and 9 months, respectively. Several factors affect the prognosis of tumors, such as TNM stage, tumor size, site, and surgical margin. A study by Lee *et al*. [17] showed that p53 protein over-expression and high Ki-67 labeling index maybe predictors of poor prognosis. Given the limited sample size in our study, we did not attempt to analyze the independent prognostic factors regarding primary gastric ASC.

## Conclusions

Primary gastric ASC is a rare entity with poor prognosis. Surgical resection is still the gold standard treatment for primary gastric ASC, although the rate of tumor recurrence/metastasis remains high even after radical resection. Adjuvant therapy (chemotherapy and/or radiotherapy) can improve survival time. Large-sample investigations are needed to determine the prognostic factors and find new therapeutic approaches to achieve better prognosis.

## Abbreviations

AC: adenocarcinoma components
ASC: adenosquamous carcinoma
CEA: carcinoembryonic antigen
CgA: chromogranin A
CK: cytokeratin
SC: squamous cell carcinoma components
Syn: synaptophysin
